# SEMA3B inhibits TGFβ-induced extracellular matrix protein production and its reduced levels are associated with a decline in lung function in IPF

**DOI:** 10.1152/ajpcell.00681.2023

**Published:** 2024-04-22

**Authors:** Dan J. K. Yombo, Sudhir Ghandikota, Chanukya P. Vemulapalli, Priyanka Singh, Anil G. Jegga, William D. Hardie, Satish K. Madala

**Affiliations:** ^1^Division of Pulmonary Medicine, Cincinnati Children’s Hospital Medical Center, Cincinnati, Ohio, United States; ^2^Department of Pediatrics, College of Medicine, University of Cincinnati, Cincinnati, Ohio, United States; ^3^Division of Biomedical Informatics, Cincinnati Children’s Hospital Medical Center, Cincinnati, Ohio, United States; ^4^Division of Pulmonary, Critical Care and Sleep Medicine, Department of Internal Medicine, University of Cincinnati College of Medicine, Cincinnati, Ohio, United States

**Keywords:** extracellular matrix, fibroblasts, pulmonary fibrosis, SEMA3B, TGFβ

## Abstract

Idiopathic pulmonary fibrosis (IPF) is marked by the activation of fibroblasts, leading to excessive production and deposition of extracellular matrix (ECM) within the lung parenchyma. Despite the pivotal role of ECM overexpression in IPF, potential negative regulators of ECM production in fibroblasts have yet to be identified. Semaphorin class 3B (SEMA3B), a secreted protein highly expressed in lung tissues, has established roles in axonal guidance and tumor suppression. However, the role of SEMA3B in ECM production by fibroblasts in the pathogenesis of IPF remains unexplored. Here, we show the downregulation of *SEMA3B* and its cognate binding receptor, neuropilin 1 (*NRP1*), in IPF lungs compared with healthy controls. Notably, the reduced expression of SEMA3B and NRP1 is associated with a decline in lung function in IPF. The downregulation of SEMA3B and NRP1 transcripts was validated in the lung tissues of patients with IPF, and two alternative mouse models of pulmonary fibrosis. In addition, we show that transforming growth factor-β (TGFβ) functions as a negative regulator of *SEMA3B* and *NRP1* expression in lung fibroblasts. Furthermore, we demonstrate the antifibrotic effects of SEMA3B against TGFβ-induced ECM production in IPF lung fibroblasts. Overall, our findings uncovered a novel role of SEMA3B in the pathogenesis of pulmonary fibrosis and provided novel insights into modulating the SEMA3B-NRP1 axis to attenuate pulmonary fibrosis.

**NEW & NOTEWORTHY** The excessive production and secretion of collagens and other extracellular matrix proteins by fibroblasts lead to the scarring of the lung in severe fibrotic lung diseases. This study unveils an antifibrotic role for semaphorin class 3B (SEMA3B) in the pathogenesis of idiopathic pulmonary fibrosis. SEMA3B functions as an inhibitor of transforming growth factor-β-driven fibroblast activation and reduced levels of SEMA3B and its receptor, neuropilin 1, are associated with decreased lung function in idiopathic pulmonary fibrosis.

## Graphical abstract

**Figure:**
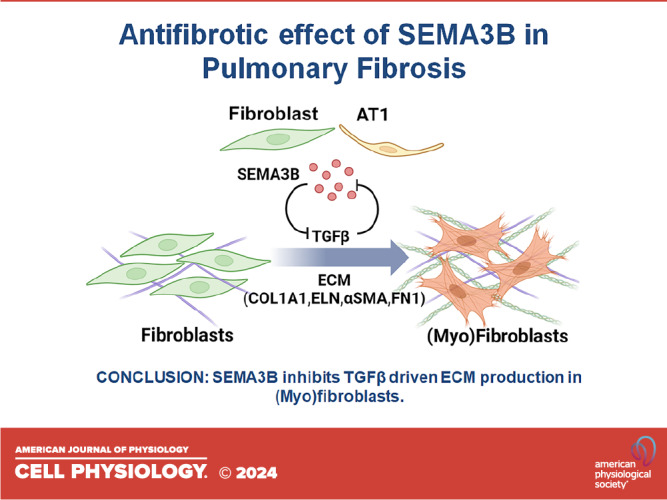

## INTRODUCTION

Idiopathic pulmonary fibrosis is a chronic and fatal interstitial lung disease characterized by a progressive decline in lung function, with the forced vital capacity (FVC) reduction exceeding 10%, and persistent accumulation of extracellular matrix (ECM), resulting in irreversible scarring in the lung parenchyma (1). Recent studies have underscored the growing prevalence and incidence of IPF in the United States and other countries (2, 3). Despite the surge in research endeavors and clinical trials directed at effective treatments, therapeutic options for patients with idiopathic pulmonary fibrosis (IPF) remain limited. Nintedanib and pirfenidone are the only two drugs approved by the Food and Drug Administration to treat IPF, and these new therapies were shown to slow the decline in lung function in IPF patients. However, they do not provide a cure or improve survival and are associated with severe side effects (4). There is an urgent need to develop novel and more effective treatments that can prevent and reverse fibrosis, improve the quality of life, and reduce mortality in patients with IPF. The development of improved antifibrotic therapies requires a further understanding of the molecular mechanisms that drive fibroblast activation and lung tissue scarring.

Fibroblasts play a major role in the process of tissue repair and wound healing following lung injury under physiological conditions, and their activation or clearance is tightly regulated by epithelium-derived profibrotic and antifibrotic molecules. In the pathogenesis of IPF, the master regulators of tissue fibrosis such as transforming growth factor-β (TGFβ) can activate fibroblasts and transform them into ECM-producing and contractile myofibroblasts leading to uncontrolled ECM production, tissue remodeling, inflammation, and the progressive scarring in the distal areas of the lung. These activated fibroblasts secrete excessive amounts of ECM components such as collagen, fibronectin, and elastin. Excessive ECM production results in stiffening of lung tissue and impaired lung function (4–7). Therefore, understanding the mechanisms underlying fibroblast activation and ECM production is important for the development of effective pulmonary fibrosis treatments. Nevertheless, the lack of knowledge regarding negative regulators of fibroblast activation capable of restraining ECM production and enhancing lung function poses a significant hurdle to the advancement of innovative antifibrotic therapies.

Semaphorins (SEMAs) are a large family of secreted or transmembrane proteins that are characterized by the presence of a “SEMA” domain, which is essential for interacting with their signaling membrane receptor plexins (8). SEMAs are key regulators of cell morphology and motility and participate in various biological processes, including immune responses and organogenesis (9, 10). SEMA3B is a secreted glycoprotein that belongs to class 3 SEMAs and is expressed in different cells of the lungs, including airway epithelial cells and fibroblasts (11). SEMA3B was first described as a negative mediator of neuronal guidance by binding to neuropilin receptors, and it has been extensively studied as a tumor suppressor protein in various tumors, including lung and breast carcinoma, through the activation of apoptosis and inhibition of migration and tumor cell growth (12, 13). Deletion or repression of SEMA3B has been observed in various cancers, including lung carcinoma, where its reexpression inhibits tumor cell growth (14, 15). SEMA3B signals via the receptor plexin A and the coreceptor neuropilin 1 or 2 (NRP1 or NRP2). NRPs are transmembrane receptors that are expressed in various cells, including immune cells, lung epithelial cells, and fibroblasts (16, 17). NRPs interact with SEMAs and other growth factors, including vascular endothelial growth factor (VEGF), TGFβ1, fibroblast growth factors, and platelet-derived growth factor-BB (PDGF); these growth factors are also known to participate in the development of pulmonary fibrosis (18–20). A recent study by Meng et al. (21) described that SEMA3F and SEMA3B proteins inhibit cell growth by activating the HIPPO pathway, and another group (22) uncovered that the expression of SEM3A prevents endothelial to mesenchymal differentiation and decreases ECM deposition in atrial fibrosis. Although studies have investigated the role of SEMA3 proteins in multiple disease conditions, the role of the SEMA3B-NRP axis in pulmonary fibrosis remains unknown.

This study investigated the role of SEMA3B signaling in the pathogenesis of idiopathic pulmonary fibrosis. The expression of SEMA3B and its coreceptor NRP1 was downregulated in the lungs and fibroblasts of patients with IPF, and their levels positively correlated with the decline in lung function. Furthermore, SEMA3B treatment of primary IPF lung fibroblasts inhibited TGFβ1-induced expression of ECM production in IPF lung fibroblasts. This study revealed a previously unrecognized role of SEMA3B in the pathogenesis of pulmonary fibrosis and provides critical insight into targeting the SEMA3B-NRP1 axis as a potential therapeutic option for pulmonary fibrosis.

## MATERIALS AND METHODS

### Human Samples

IPF and healthy lung samples were obtained with the assistance of the Translational Pulmonary Science Center, University of Cincinnati Medical Center. The Translational Pulmonary Science Center collects and maintains a repository of tissue and primary cells from patients with chronic lung diseases. The local University of Cincinnati Institutional Review Board (IRB 2013-8157) reviews the procedures in place to ensure adequate protection of human subjects and protection of patient privacy and confidentiality. Donor lung samples with no lung diseases were used as normal lung biopsies and all materials were de-identified to the research team.

### Mouse Models of TGFα- and Bleomycin-Induced Pulmonary Fibrosis

We used total lung RNA isolated from the lungs of two experimental animal models of pulmonary fibrosis to evaluate the expression of SEMA3B and NRP1. In the bleomycin model, mice were treated with repetitive intradermal injections of bleomycin (BLM) to induce pulmonary fibrosis as previously described (23, 24). Briefly, C57BL/6 male and female mice at 12 wk of age were treated with 6 U of bleomycin in 50 µL of saline in the dorsal region daily for 5 days per week for 4 weeks. On day 28, mice were euthanized, and lungs were collected for further analysis. Control mice were treated with the saline solution following the same procedure. In the TGFα model of pulmonary fibrosis, we used TGFα-overexpressing mice of FVB/NJ inbred-strain background as previously described (25, 26). Homozygous CCSPrtTA mice were mated with heterozygous (TetO) 7-cmv TGF-α mice to generate bitransgenic mice TGF-α (CCSP/TGF-α) mice and littermate controls (CCSP/-). Both male and female mice aged 10–16 wk were used in the experiment. To induce TGFα expression, doxycycline (DOX; Millipore Sigma) was administered in food (62.5 mg/kg), which caused the mice to develop severe fibrotic lung disease. Mice were maintained on the DOX regimen for up to 8 wk before euthanization. All mice were housed under specific pathogen-free conditions at the Cincinnati Children’s Hospital Medical Center, a medical facility approved by the American Association for the Accreditation of Laboratory Animal Care. All experimental procedures were approved by the Animal Care and Use Committee of Cincinnati Children’s Hospital Medical Center.

### IPF Transcriptomic Datasets

Publicly available microarray lung transcriptomic datasets were obtained from the NCBI Gene Expression Omnibus (GEO) repository. The lung Tissue Research Consortium data set GSE47460 containing expression profiles and clinical attributes for 160 IPF patients and 108 healthy controls was used to determine the expression of SEMA3B and NRP1 and their correlation with the predicted forced expiratory volume in 1 s (FEV_1_), forced vital capacity (FVC), and the diffusing capacity of the lungs for carbon monoxide (DL_CO_) as previously described (27, 28). In addition, we used an independent IPF cohort, GSE150910 containing lung RNA-sequencing (RNA-seq) data from patients with IPF and healthy controls (*n* = 40–60/group) (29). Differentially expressed genes in IPF compared with healthy controls from these datasets were identified as described in our previous publication (28).

### Single Cell RNA-Seq Analysis

LungMap (www.lungmap.net) was used to generate the UMAP of healthy human lung cells, where “Human CellCards Multi-Study CellRef 1.0 Atlas” data set was accessed using Human Lung CellRef (v1) ShinyCell Browser, and cell type annotation “celltype level 1” was set for the final images. For IPF versus non-IPF scRNA-seq analysis, we have used the publicly available GSE136831 data set from GEO database and analyzed using Seurat (v4.3.0) in R-studio (v2023.06.0) (30). Processed datasets were loaded into Seurat Object, and cell types were defined using cell annotations provided in the metadata of the data set.

### Immunohistochemistry Staining

Formalin-fixed and paraffin-embedded human lung tissue sections of IPF (*n* = 6) and healthy (*n* = 5) controls were probed with antibodies against SEMA3B (Novus Biologicals) or NRP1 (Proteintech) for immunohistochemistry staining as previously described (31, 32). We used goat anti-rabbit IgG as an isotype control antibody and observed no detectable staining (data not shown). Hematoxylin counterstaining was used to stain nuclei in the color blue. All images were obtained using a Keyence BZ-X800 microscope at high magnification (×63).

### Primary Fibroblast Cultures and Treatments

Primary fibroblasts from IPF and healthy control lung tissues were isolated using collagenase digestion as previously described (33, 34). Fibroblasts were cultured in DMEM supplemented with 10% FBS and antibiotics, and the fibroblasts used in the experiments were between passages 1 and 3. Fibroblasts were treated overnight with TGFβ1 (20 ng/mL), recombinant SEMA3B (200 or 400 ng/mL), TGFα (100 ng/mL), or connective tissue growth factor (CTGF; 50 ng/mL) in DMEM media containing low-serum (0.5% FCS). To assess the effect of SEMA3B on TGFβ1-driven ECM gene expression, fibroblasts were treated with media, SEMA3B, and/or TGFβ1 for 16 h, and RT-PCR was performed to quantify transcripts or treated for 72 h to quantify proteins using Western blots.

### RNA Preparation and Real-Time PCR

Lung tissues were homogenized using beads and a high-speed tissue homogenizer (ThermoFisher) to isolate RNA from lung tissue homogenates using an RNAeasy mini kit (Qiagen) as previously described (23, 35). cDNA was synthesized using Superscript III (Invitrogen), and real-time PCR was performed using SYBR Green Select Master Mix (Applied Biosystems, Foster City, CA) and a CFX Opus 384 Touch Real-Time PCR instrument (Bio-Rad, Hercules, CA). The relative expression of genes of interest was analyzed using CFX Maestro software version 4.0. The target gene transcripts were normalized to human β-actin or hypoxanthine-guanine phosphoribosyl transferase (HPRT) for human and mouse samples, respectively. The primer list of genes used in this study is provided in Table 1.

**Table 1.** List of primers used for RT-PCR

Gene ID	Forward Primer	Reverse Primer
hβ-ACTIN	CCA ACC GCG AGA AGA TGA	CCA GAG GCG TAC AGG GAT AG
hCOL1	GGGATTCCCTGGACCTAAAG	GGAACACCTCGCTCTCCA
hCOL3	CTGGACCCCAGGGTCTTC	CATCTGATCCAGGGTTTCCA
hELN	GCC ATT CCT GGT GGA GTT CCT GGA	ACC GCA CCT GCA GAC ACT CCT AAG
hFN1	CTGGCCGAAAATACATTGTAAA	CCACAGTCGGGTCAGGAG
hSEMA3B	CGTCCTCTTCATTGGCACA	GCCGAGTCCTCAAACACG
hSMA	GCTTTCAGCTTCCCTGAACA	GGAGCTGCTTCACAGGATTC
hNRP1	TACCCTGAGAATGGGTGGAC	CGTGACAAAGCGCAGAAG
hNRP2	GGACCCCCAACTTGGATT	ATGGTTAAAAAGCGCAGGTC
mHPRT	GCCCTTGACTATAATGAGTACTTCAGG	TTCAACTTGCGCTCATCTTAGG
mNRP1	TCTGAGATAATCCTGGAGTTTGAA	CCAATGTGAGGGCCAACTT
mNRP2	ATGGCTGGACACCCAATTT	ATGGTTAGGAAGCGCAGGT
mSEMAB	TTCTTCTTCCGCGAGTCC	GCCACCCAGGTCATTCCT

COL1, collagen 1; ELN, elastin; SMA, smooth muscle actin; FN1, fibronectin 1; SEMAB, semaphorin class 3B; NPR1, neuropilin 1; HPRT, hypoxanthine-guanine phosphoribosyl transferase; m, mouse; h, human.

### Western Blot Analysis

Total cell lysates were prepared from lung fibroblasts treated with media, SEMA3B, and/or TGFβ1 for 72 h, and immunoblotting was performed as previously described (24). In brief, the cell lysates were electrophoresed in reducing conditions and then transferred onto a nitrocellulose membrane. The membranes were blocked with 5% BSA in 0.1% Tween phosphate buffer saline for 1 h at room temperature and incubated with primary antibodies overnight at 4°C. The primary antibodies used included anti-GAPDH (1:2,000; Bethyl Labs), anti-collagen 1 (COL1; 1:1,000; CST); anti-elastin (anti-ELN; 1:1,000, Proteintech), anti-fibronectin 1 (anti-FN1; 1:10,00; Santa Cruz Technology), and anti-α-smooth muscle actin (anti-αSMA; 1:20,000; Millipore Sigma). The membranes were washed with Tween phosphate buffer saline and incubated with appropriate secondary antibodies conjugated with peroxidase for 1 h. ECL Prime Western Blotting Detection Reagent (ThermoFisher Scientific) was used to visualize antibodies. The densities of the bands on the membranes were scanned and analyzed using the LCD4000 imaging camera and PhosphoImager Multi Gage software (Fujifilm, Valhalla, NY). The expression of proteins of interest was normalized to GAPDH.

### Statistical Analysis

All data were analyzed using Prism (version 9; GraphPad, San Diego, CA) R statistical software (RCRAN). Pearson’s *r* test was used for linear correlation analysis between SEMA3B or NRP1 expression and percent FEV1, FVC, or percent DL_CO_, and graphs were generated using R software and plugins. Quantitative data are presented as violin plots (transcriptomics data) or bars with means ± SE Unpaired two-tailed *t* test and one-way ANOVA with post hoc Tukey’s test were used to compare the means between two or multiple experimental groups, respectively. *P* < 0.05 was considered statistically significant.

## RESULTS

### Reduced SEMA3B and NRP1 Transcript Levels Correlate with Lung Function Decline in IPF

**Figure 1.**
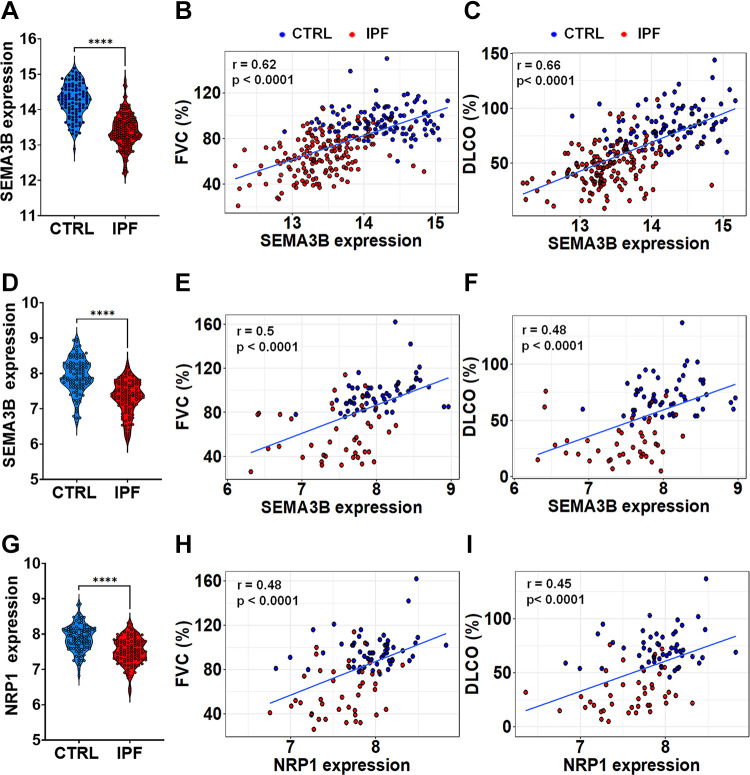
Reduced semaphorin class 3B (SEMA3B) and neuropilin 1 (NRP1) levels associate with lung function loss in idiopathic pulmonary fibrosis (IPF). *A*: SEMA3B transcript levels were lower in patients with IPF (*n* = 160) compared to healthy subjects (*n* = 108) based on the RNA microarray dataset GSE47460. ****P < 0.0001. *B* and *C*: GSE47460 data show a positive correlation between SEMA3B transcript levels and both percent forced vital capacity (FVC) and diffusing capacity of the lungs for carbon monoxide (DL_CO_) in both IPF (red) and healthy subjects (blue). The blue line represents the linear correlation. *D*: RNA-sequencing data from GSE150910 confirms decreased SEMA3B transcript levels in IPF lungs compared to healthy controls (CTRL; *n* = 40–60/group). ****P < 0.0001. *E* and *F*: similar to GSE47460, GSE150910 data show a positive correlation between SEMA3B transcript levels and percent FVC or DL_CO_ in IPF (red) and healthy subjects (blue). *G*: NRP1 transcript levels were also reduced in IPF lungs compared to healthy lungs according to the RNA-sequencing data from GSE150910. ****P < 0.0001. *H* and *I*: a positive correlation between NRP1 transcript levels and both percent FVC and DL_CO_ is observed in IPF (red) and healthy subjects (blue) from the GSE150910 dataset. The correlation coefficient (*r*) and *P* value are displayed within the scatter plots.

Our published study using gene set enrichment analysis (GSEA) identified several important molecular regulators within gene modules closely linked to disease severity in the IPF data set, GSE47460 (28). Notably, the transcript levels of *SEMA3B* in the lungs of patients with IPF were significantly reduced compared with those in healthy controls (Fig. 1*A*). Intriguingly, this diminished SEMA3B expression exhibited a robust positive correlation (*r* = 0.62) with the decline in lung function, as assessed by forced vital capacity (FVC). Similarly, SEMA3B expression displayed a positive correlation (*r* = 0.66) with the reduction in the diffusing capacity of the lungs for carbon monoxide (%DL_CO_) (Fig. 1, *B* and *C*). To further substantiate these findings, we extended our analysis to an independent data set of lung RNAseq data, GSE150910, comprising IPF and healthy control subjects (29). In line with our initial data set, SEMA3B transcript levels exhibited significant downregulation in the lungs of IPF patients when compared with healthy controls (Fig. 1*D*). Correspondingly, SEMA3B transcript levels displayed positive correlations with lung function decline in IPF patients, yielding correlation coefficients of *r* = 0.50 and *r* = 0.46 when associated with percent FVC and percent DL_CO_ values, respectively (Fig. 1, *E* and *F*). As SEMA class 3 proteins, including SEMA3B, necessitate the presence of coreceptor moieties NRP1 or NRP2 before interacting with their signal transducing receptors plexins A1-4, we assessed whether the expression of the SEMA3B coreceptor NRP1 and NRP2 was also perturbed in the lungs of IPF compared with those of healthy controls (36). Our analysis of IPF gene expression datasets revealed a significant reduction in NRP1 transcript levels in the lungs of IPF patients compared to those of healthy control subjects. Our findings in Supplemental Fig. S1 (all Supplemental material is available at https://doi.org/10.6084/m9.figshare.25432315) showed no significant correlation between NRP2 expression and lung function decline in either IPF patients or healthy controls. Conversely, the downregulation of SEMA3B and NRP1 is associated with the progressive decline in lung function observed in IPF patients (Fig. 1, *H* and *I*). These findings highlight the potential importance of SEMA3B and NRP1 as key molecular players in IPF pathogenesis.

### Downregulation of SEMA3B and NRP1 in IPF Lungs

**Figure 2.**
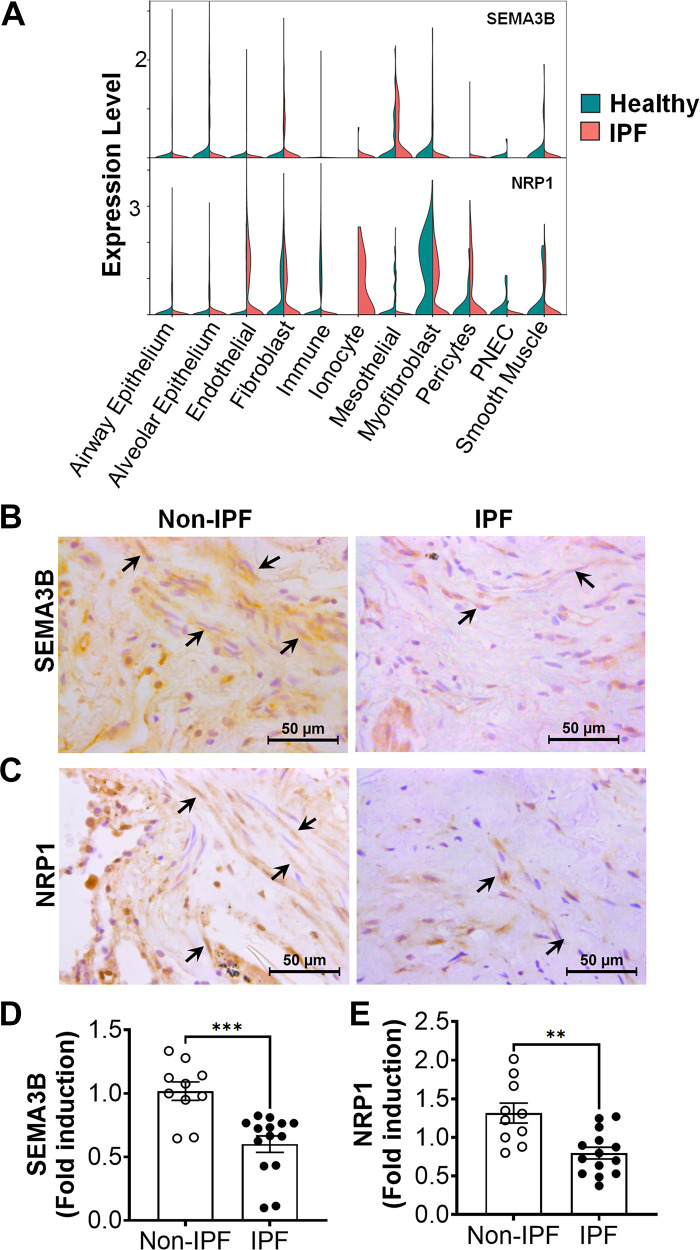
Downregulation of semaphorin class 3B (SEMA3B) and neuropilin 1 (NRP1) in idiopathic pulmonary fibrosis (IPF) lungs. *A*: violin plot showing the expression levels of SEMA3B and NRP1 across various cell types in non-IPF and IPF lungs. Data are from a publicly available dataset (GSE136831) for non-IPF and IPF lung scRNA-seq. *B* and *C*: representative immunohistochemical images of distal lung biopsies from normal and IPF lungs stained with antibodies against SEMA3B (*B*) and NRP1 (*C*). Scale bar = 50 μm. Arrows highlight spindle-shaped mesenchymal cells positive for SEMA3B or NRP1 (*n* = 5 per group). *D* and *E*: transcript levels of SEMA3B and NRP1 were quantified in fibroblasts isolated from the lungs of IPF patients and healthy controls using RT-PCR. Student’s *t* test was performed with *n* = 10–14 per group. ***P < 0.001, **P < 0.01.

To identify lung cells involved in the expression of SEMA3B and NRP1, we analyzed scRNA-seq datasets from the LungMAP Consortium that comprises 505,256 cells from 148 normal human lung samples. We observed that SEMA3B is predominantly expressed by mesenchymal cells including alveolar fibroblast 1 (AF1), alveolar fibroblast 2 (AF2), vascular and airway smooth muscle cells, endothelial cells (CAP1 and CAP2), and AT1 epithelial cells (Supplemental Fig. S2). Conversely, NRP1 is expressed by mesenchymal cells (AF1, CAP1, and CAP2), macrophages, and AT2 epithelial cells (Supplemental Fig. S2). Also, we compared the cell-specific expression of SEMA3B and NRP1 in IPF compared to non-IPF lung cells in single-cell RNA seq datasets (30). Notably, we observed reduced expression of SEMA3B and NRP1 in myofibroblasts of IPF compared to healthy controls (Fig. 2*A*). Further we conducted immunohistochemical staining of SEMA3B and NRP1 proteins in lung tissue sections obtained from IPF patients and healthy controls (Fig. 2, *B* and *C*). We observed robust staining of SEMA3B and NRP1 proteins in lung sections from healthy control subjects, prominently localized in spindle-shaped mesenchymal cells. However, a marked reduction in the SEMA3B signal was evident in IPF lung sections compared with healthy controls. Notably, this decreased expression of SEMA3B was particularly pronounced in spindle-shaped mesenchymal cells (Fig. 2*B*). Similarly, the NRP1 signal was prominently detected in lung sections from healthy controls, inclusive of mesenchymal cells. However, in contrast, the expression of NRP1 was significantly diminished in the lungs of IPF patients (Fig. 2*C*). It is noteworthy that lung fibroblasts serve as the principal source responsible for the production and deposition of extracellular matrix (ECM) components within the lung interstitium during the progression of pulmonary fibrosis.

Given the compelling findings from the immunohistochemical staining of lung sections, which demonstrated a substantial decrease in SEMA3B and NRP1 expression in spindle-shaped mesenchymal cells within IPF lungs, we extended our investigation to assess their expression in primary lung fibroblasts obtained from both IPF patients and control subjects. Consistently, SEMA3B transcript levels were significantly downregulated in IPF lung fibroblasts when compared to those derived from healthy controls (Fig. 2*D*). In concordance, the expression of NRP1 transcript was notably reduced in primary IPF lung fibroblasts relative to their counterparts from control subjects (Fig. 2*E*). Thus our comprehensive analyses establishes that the downregulation of SEMA3B and its coreceptor NRP1 manifests at both the transcriptional and protein levels within the lungs and fibroblasts of IPF patients. These observations underscore the significance of SEMA3B and NRP1 as pivotal regulators of IPF pathogenesis.

### TGFα and TGFβ1 Regulate the Expression of SEMA3B in Pulmonary Fibrosis

**Figure 3.**
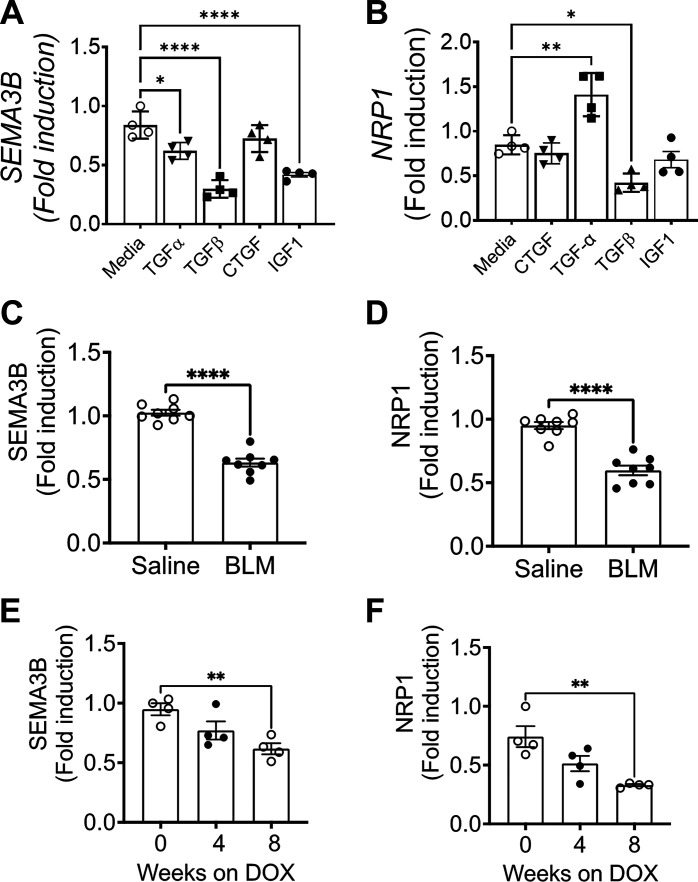
Downregulation of semaphorin class 3B (SEMA3B) and neuropilin 1 (NRP1) in mouse models of pulmonary fibrosis. *A* and *B*: quantification of SEMA3B and Nrp1 transcripts in fibroblasts that were treated with profibrotic growth factors connective tissue growth factor (CTGF; 100 ng/mL), transforming growth factor-α (TGFα; 100 ng/mL), TGFβ1 (20 ng/mL), and IGF-1 (20 ng/mL) for 16 h using RT-PCR with *n* = 4/group, Data are shown as means ± SE. One-way ANOVA was used. *P < 0.05. *C* and *D*: SEMA3B and NRP1 transcripts were measured using RT-PCR in the lungs of mice treated with saline or bleomycin (BLM) for 4 weeks. Student’s *t* test was performed with *n* = 8/group. ****P < 0.0001. *E* and *F*: SEMA3B and NRP1 transcripts were measured using RT-PCR in the lungs of TGFα transgenic mice on doxycycline (DOX) food for 0, 4, and 8 wk. One-way ANOVA was used. Data are shown as means ± SE. **P < 0.01.

The role of various growth factors and cytokines in driving fibrotic processes across different organs, including the lungs, is well-established (37, 38). Among these, TGFα and TGFβ1 have been shown to function as pivotal profibrotic growth factors with evidence of their involvement in fibroblast activation and the development of pulmonary fibrosis in both human subjects and animal models (18, 39, 40). To identify the profibrotic growth factors responsible for the downregulation of SEMA3B, we conducted experiments involving primary lung fibroblasts isolated healthy lungs. Normal lung fibroblasts were treated with several profibrotic growth factors, including TGFα, TGFβ1, IGF1, and CTGF. Notably, our findings revealed a significant reduction in SEMA3B expression specifically in normal lung fibroblasts when treated with TGFβ1, TGFα, and IGF1, as compared to media-treated controls, whereas CTGF had limited or no effect on SEMA3B expression (Fig. 3*A*). Similarly, Nrp1 expression was significantly reduced with TGFβ1 compared to CTGF, IGF1, or media treatment in normal lung fibroblasts (Fig. 3*B*). Intriguingly, TGFα treatment resulted in increased expression of Nrp1 compared to media treated controls (Fig. 3*B*). These results indicate that TGFβ1 as the principal profibrotic growth factor contributing to the reduced expression of SEMA3B and Nrp1 in the pathogenesis of pulmonary fibrosis.

To determine the role of the SEMA3B-NRP1 axis in preclinical mouse models of pulmonary fibrosis, we quantified the expression of SEMA3B and NRP1 in the lungs of mice with severe fibrotic lung disease in two murine models of pulmonary fibrosis, bleomycin (BLM)-induced, and TGFα-induced lung fibrosis (23, 41). Our published studies have shown that repetitive intradermal administration of BLM induces severe fibrotic lung disease in mice with a significant decline in lung function (23, 24). Similar to the findings in IPF lungs, we observed a significant decline in the expression of SEMA3B in the lungs of BLM-treated mice compared to saline-treated control mice (Fig. 3*C*). Similarly, the transcript levels of *NRP1* were also downregulated in the lungs of BLM-treated mice (Fig. 3*D*). Furthermore, we also used another model of lung fibrosis in which epithelial overexpression of TGFα induces spontaneous pulmonary fibrosis (TGFα^OE^-induced pulmonary fibrosis) (42, 43). The expression of *SEMA3B* and *NRP1* was measured in the lungs of these fibrotic mice following 4 and 8 wk of doxycycline (DOX) exposure. Transcript levels of *SEMA3B* were decreased in a time-dependent manner with a significant decrease at 8 wk of DOX exposure in fibrotic mice compared to control mice not exposed to the DOX regimen (Fig. 3*E*). Additionally, *NRP1* transcript expression was decreased in the lungs of TGFα-overexpressing mice compared to their controls (Fig. 3*F*). These data using IPF samples and alternative preclinical models suggest that SEMA3B and NRP1 are downregulated in the pathogenesis of pulmonary fibrosis.

### SEMA3B Attenuates the TGFβ1-Driven ECM Production

**Figure 4.**
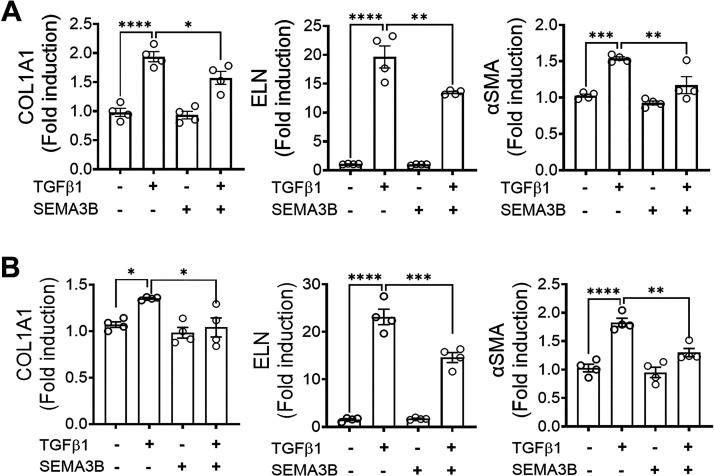
Semaphorin class 3B (SEMA3B) inhibits TGFβ1-driven extracellular matrix (ECM) gene expression in fibroblasts. *A*: upregulation of the transcripts of ECM genes [collagen 1A1 (COL1A1), elastin (ELN), and α-smooth muscle actin (αSMA)] in normal lung fibroblasts treated with media, transforming growth factor-β (TGFβ), SEMA3B, or both TGFβ and SEMA3B for 16 h. *B*: upregulation of the transcripts of ECM genes (COL1A1, ELN, and αSMA) in IPF fibroblasts treated with media, TGFβ, SEMA3B, or both TGFβ and SEMA3B for 16 h. Data are shown as the means ± SE. Student’s *t* test was performed with *n* = 4/group. *P < 0.05, **P < 0.01, ***P < 0.001, ****P < 0.0001.

**Figure 5.**
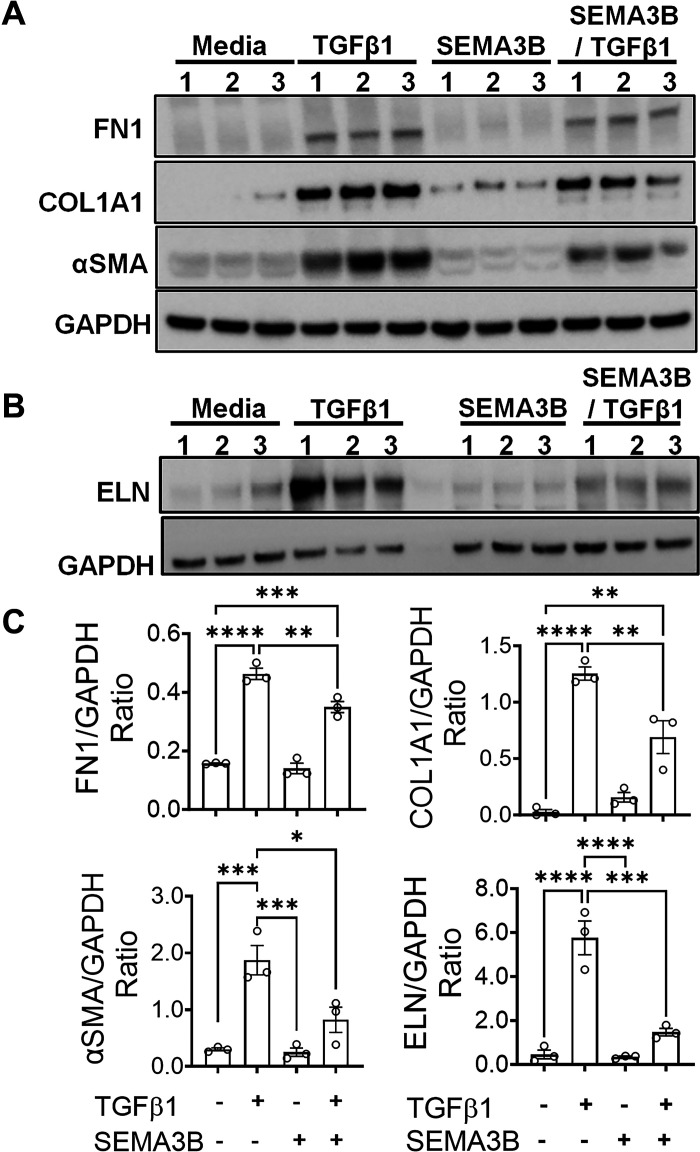
Semaphorin class 3B (SEMA3B) inhibits the transforming growth factor-β1 (TGFβ1)-driven extracellular matrix (ECM) protein production in Idiopathic pulmonary fibrosis (IPF) lung fibroblasts. *A*: IPF lung fibroblasts were treated with media, SEMA3B (400 ng/mL), and/or TGFβ1 (20 ng/mL) for 72 hours, and cell lysates were immunoblotted with antibodies against collagen 1 (COL1), fibronectin 1 (FN1), α-smooth muscle actin (αSMA), and GAPDH. *B*: IPF lung fibroblasts were treated with media, SEMA3B (400 ng/mL), and/or TGFβ1 (20 ng/mL) for 72 h, and cell lysates were immunoblotted with antibodies against elastin (ELN) and GAPDH. *C*: densitometric quantification of COL1, FN1, αSMA, and ELN protein levels normalized to GAPDH. Data are shown as the means ± SE. A one-way ANOVA test was performed with *n* = 3/group. *P < 0.05, **P < 0.01, ***P < 0.001, ****P < 0.0001.

To determine the effect of TGFβ1 on ECM production in fibroblasts, fibroblasts isolated from healthy and IPF lungs were treated with TGFβ1, and the expression of ECM gene transcripts was measured using RT-PCR. As expected, TGFβ1 induced the expression of COL1, ELN, and αSMA transcript levels in both normal and IPF lung fibroblasts compared to media-treated fibroblasts (Fig. 4, *A* and *B*). To test whether SEMA3B alters TGFβ1-induced ECM production, fibroblasts were treated with TGFβ1 in the presence or absence of SEMA3B. Notably, the presence of SEMA3B was sufficient to attenuate the increased expression of COL1, ELN, and αSMA ECM gene transcripts by TGFβ1 in both healthy controls and IPF lung fibroblasts (Fig. 4, *A* and *B*). To establish a negative regulation of TGFβ1-induced ECM protein production by SEMA3B, we measured the protein levels of COL1, ELN, FN1, and αSMA in cell lysates of IPF fibroblasts treated with TGFβ1 in the presence or absence of SEMA3B. TGFβ1 treatment resulted in a significant increase in the expression of COL1, ELN, FN1, and αSMA in IPF fibroblasts compared to media-treated fibroblasts. Notably, the presence of SEMA3B was sufficient to attenuate the TGFβ1-induced ECM protein production including, COL1, ELN, FN1, and αSMA in IPF fibroblasts (Fig. 5). SEMA3B alone had limited or no significant effect on the expression of ECM proteins as their levels were similar compared to media treated IPF fibroblasts (Fig. 5). These findings indicate that SEMA3B inhibits the effects of TGFβ1-induced ECM production by IPF fibroblasts. Together, our findings suggest that SEMA3B is a negative regulator of TGFβ1-induced ECM production in IPF fibroblasts.

## DISCUSSION

In this study, we investigated the expression of SEMA3B in the lungs of IPF patients and murine models of pulmonary fibrosis and determined its potential role in the pathogenesis of pulmonary fibrosis. The levels of SEMA3B and NRP1 was decreased in lung tissues and fibroblasts in IPF. Notably, there was a positive correlation between lung function and the transcript levels of SEMA3B and NRP1, suggesting an antifibrotic role for the SEMA3B-NRP1 axis and also as a potential biomarker of disease severity (44). The major profibrotic growth factor TGFβ1 downregulated the expression of SEMA3B and the coreceptor NRP1. Conversely, exogenous administration of SEMA3B was sufficient to attenuate TGFβ1-induced ECM protein production in IPF fibroblasts. This study has uncovered a previously unidentified role for the SEMA3B-NRP1 axis as a negative regulator of pulmonary fibrosis via inhibiting ECM production in fibroblasts.

Using two independent datasets of IPF lung transcriptomics, we showed downregulation of SEMA3B and NRP1 in the lungs of IPF patients. In addition to IPF, a recent study by Pinezich et al. (45) also reported decreased expression of SEMA3B protein in the lungs of cystic fibrosis patients, another pulmonary disease with extensive ECM deposition, and a progressive decline in lung function (46). In this study, we demonstrated the decreased expression of SEMA3B in IPF lungs and fibroblasts. The decreased expression of SEMA3B is not limited to IPF, we observed reduced expression of SEMA3B and NRP1 in two alternative mouse models of pulmonary fibrosis.

Our new findings revealed that multiple pro-fibrotic growth factors including TGFβ1, TGFα, and IGF1 function as negative regulators of SEMA3B expression in IPF lung fibroblasts. Similarly, TGFβ1 was effective in inhibiting NRP1 expression in IPF fibroblasts. This finding is consistent with reduced expression of SEMA3B in the lungs during TGFα and bleomycin-induced pulmonary fibrosis. Further, published studies from our laboratory and others showed increased expression and activity of TGFβ signaling during TGFα and bleomycin-induced pulmonary fibrosis. However, the present study has not investigated molecular mechanisms by which TGFβ1 downregulated the expression of SEMA3B or NRP1 in IPF fibroblasts. Published studies show hypermethylation of the promotor region and gene silencing as a potential mechanism underlying the repression of the SEMA3B expression in most malignancies, including lung adenocarcinoma (13, 47). In a recent study, the investigators found that TGFβ1 downregulated the expression of SEMA3A by upregulating the expression of miR-181b microRNA, which targets the 3′-untranslated region of SEMA3A degradation (22). Lai et al. (22) investigated the role of SEMA3A in the development of atrial fibrosis and described the inhibitory effect of SEMA3A on TGFβ1-induced endothelial-mesenchymal transition during endocardial fibrosis. Therefore, future studies are warranted to investigate how SEMA3B is downregulated by TGFβ signaling and potential microRNAs in IPF fibroblasts and also the mouse models of pulmonary fibrosis in vivo.

Previous studies have shown that TGFβ1 can also signal through the NRP receptors in a Smad2-independent manner, whereas NRP1 can bind and activate the latent form of TGFβ1 or directly interact with the active form of TGFβ1 in multiple disease conditions, including malignancy (16, 17). In addition to SEMA3 family proteins, NRP1 is a multifunctional receptor protein that can also bind to other ligands and profibrotic growth factors, including PDGFA, VEGF, and hepatocyte growth factor. Therefore, it is possible that these profibrotic factors can interfere with the binding of SEMA3B to NRP1 during pulmonary fibrosis development (18–20, 48). In addition to SEMA3B, our data showed that TGFβ1 downregulated the expression of NRP1 in IPF fibroblasts. Similarly, a study by Schrameck and colleagues (20) explored the expression of NRPs in fibrotic kidney disease and showed that TGFβ1 downregulates the expression of NRP1 in fibrotic renal tissues. Conversely, other studies have shown that NRP1 is upregulated and contributes to the activation of hepatic stellate cells and the development of liver fibrosis (49, 50). The dissimilarity in NRP1 expression in fibrotic diseases suggests that other ligands of NRP1 might be playing a role in a tissue-specific manner (19). Nevertheless, the current study demonstrates the molecular interactions between TGFβ1 and the SEMA3B-NRP1 axis in altering the expression of ECM production and pulmonary fibrosis.

Previous studies have reported the association of other SEMA family proteins with fibrotic lung diseases (10). SEMA7A is required for TGFβ1-induced pulmonary fibrosis. The loss of SEMA7A has been shown to attenuate the development of pulmonary fibrosis in a murine model of bleomycin-induced pulmonary fibrosis (51, 52). SEMA4A is another SEMA protein reported to play an important role in IPF through the induction of αSMA expression in fibroblasts (53). However, the current study has established a strong association between SEMA3B and the lung function decline in IPF. Further, the inhibitory effects of SEMA3B against TGFβ-driven ECM production in fibroblasts support the potential antifibrotic role for SEMA3B in IPF. In addition to fibroblasts, SEMA3B and NRP1 are expressed in the lungs by other cells, including airway epithelial cells ATI, ATII, endothelial, and transitional alveolar epithelial cells (11). This study focused on the role of the SEMA3B-NRP1 axis in lung fibroblasts. However, the effects of the SEMA3B-NRP1 axis may not be limited to fibroblasts and it is also possible that the effects of SEMA3B in alveolar epithelial cells and their interaction with lung fibroblasts. Future studies are needed to decipher the role of the SEMA3B-NRP1 axis using cell-specific loss of function and gain of function pre-clinical models.

This study identified a novel role of SEMA3B as a negative regulator of ECM production in fibroblasts. Supporting SEMA3B effects on ECM production, the decreased expression of SEMA3B or NRP1 is associated with reduced lung function in two independent IPF cohorts. Overall, our new findings highlight a potential antifibrotic role for SEMA3B and support modulating the SEMA3B-NRP1 axis as an innovative therapeutic approach to inhibit pulmonary fibrosis.

### Perspectives and Significance

This study identified a novel role of SEMA3B as a negative regulator of ECM production in fibroblasts. In support, the decreased expression of SEMA3B or NRP1 is associated with reduced lung function in two independent IPF cohorts. Overall, our new findings highlight an antifibrotic role for SEMA3B and support modulating the SEMA3B-NRP1 axis as an innovative therapeutic approach to inhibit pulmonary fibrosis.

## DATA AVAILABILITY

The data that support the findings of this study will be made available upon reasonable request to the corresponding author.

## SUPPLEMENTAL MATERIAL

10.6084/m9.figshare.25432315 Supplemental Figs. S1 and S2: https://doi.org/10.6084/m9.figshare.25432315.

## GRANTS

This study was supported in part by the Cincinnati Children’s Hospital and Medical Center (to W.D.H.) and National Heart, Lung, and Blood Institute Grants 1R01 HL134801 and 1R01 HL157176 (to S.K.M.).

## DISCLOSURES

A.G.J. is a member of the Scientific Advisory Board of Gen1E Lifesciences. The other authors declare no conflicts of interest.

## AUTHOR CONTRIBUTIONS

D.J.K.Y., A.G.J., W.D.H., and S.K.M. conceived and designed research; D.J.K.Y., S.G., C.P.V., P.S., A.G.J., and S.K.M. performed experiments; D.J.K.Y., S.G., C.P.V., P.S., A.G.J., and S.K.M. analyzed data; D.J.K.Y., S.G., C.P.V., P.S., A.G.J., W.D.H., and S.K.M. interpreted results of experiments; D.J.K.Y., C.P.V., P.S., A.G.J., and S.K.M. prepared figures; D.J.K.Y. and S.K.M. drafted manuscript; D.J.K.Y., S.G., C.P.V., P.S., A.G.J., W.D.H., and S.K.M. edited and revised manuscript; D.J.K.Y., S.G., C.P.V., P.S., A.G.J., W.D.H., and S.K.M. approved final version of manuscript.

## References

[1] Raghu G, Collard HR, Egan JJ, Martinez FJ, Behr J, Brown KK, , et al An official ATS/ERS/JRS/ALAT statement: idiopathic pulmonary fibrosis: evidence-based guidelines for diagnosis and management. Am J Respir Crit Care Med 183: 788–824, 2011. doi:10.1164/rccm.2009-040GL. 21471066 PMC5450933

[2] Kaul B, Lee JS, Zhang N, Vittinghoff E, Sarmiento K, Collard HR, Whooley MA. Epidemiology of idiopathic pulmonary fibrosis among U.S. Veterans, 2010-2019. Ann Am Thorac Soc 19: 196–203, 2022. doi:10.1513/AnnalsATS.202103-295OC. 34314645 PMC8867365

[3] Kondoh Y, Suda T, Hongo Y, Yoshida M, Hiroi S, Iwasaki K, Takeshima T, Homma S. Prevalence of idiopathic pulmonary fibrosis in Japan based on a claims database analysis. Respir Res 23: 24, 2022. doi:10.1186/s12931-022-01938-6. 35135550 PMC8822670

[4] Sontake V, Gajjala PR, Kasam RK, Madala SK. New therapeutics based on emerging concepts in pulmonary fibrosis. Expert Opin Ther Targets 23: 69–81, 2019. doi:10.1080/14728222.2019.1552262. 30468628 PMC6294998

[5] Meyer KC. Pulmonary fibrosis, part I: epidemiology, pathogenesis, and diagnosis. Expert Rev Respir Med 11: 343–359, 2017. doi:10.1080/17476348.2017.1312346. 28345383

[6] Stancil IT, Michalski JE, Schwartz DA. An airway-centric view of idiopathic pulmonary fibrosis. Am J Respir Crit Care Med 206: 410–416, 2022. doi:10.1164/rccm.202109-2219PP. 35446237 PMC12039158

[7] King TE Jr, Pardo A, Selman M. Idiopathic pulmonary fibrosis. Lancet 378: 1949–1961, 2011. doi:10.1016/S0140-6736(11)60052-4. 21719092

[8] Nakamura F, Kalb RG, Strittmatter SM. Molecular basis of semaphorin-mediated axon guidance. J Neurobiol 44: 219–229, 2000. doi:10.1002/1097-4695(200008)44:2<219::AID-NEU11>3.0.CO;2-W. 10934324

[9] Alto LT, Terman JR. Semaphorins and their signaling mechanisms. Methods Mol Biol 1493: 1–25, 2017. doi:10.1007/978-1-4939-6448-2_1. 27787839 PMC5538787

[10] Movassagh H, Khadem F, Gounni AS. Semaphorins and their roles in airway biology: potential as therapeutic targets. Am J Respir Cell Mol Biol 58: 21–27, 2018. doi:10.1165/rcmb.2017-0171TR. 28817310

[11] Marconett CN, Zhou B, Sunohara M, Pouldar TM, Wang H, Liu Y, Rieger ME, Tran E, Flodby P, Siegmund KD, Crandall ED, Laird-Offringa IA, Borok Z. Cross-species transcriptome profiling identifies new alveolar epithelial type I cell-specific genes. Am J Respir Cell Mol Biol 56: 310–321, 2017. doi:10.1165/rcmb.2016-0071OC. 27749084 PMC5359537

[12] Tang H, Wu Y, Liu M, Qin Y, Wang H, Wang L, Li S, Zhu H, He Z, Luo J, Wang H, Wang Q, Luo S. SEMA3B improves the survival of patients with esophageal squamous cell carcinoma by upregulating p53 and p21. Oncol Rep 36: 900–908, 2016. doi:10.3892/or.2016.4901. 27349960

[13] Loginov VI, Dmitriev AA, Senchenko VN, Pronina IV, Khodyrev DS, Kudryavtseva AV, Krasnov GS, Gerashchenko GV, Chashchina LI, Kazubskaya TP, Kondratieva TT, Lerman MI, Angeloni D, Braga EA, Kashuba VI. Tumor suppressor function of the SEMA3B gene in human lung and renal cancers. PLoS One 10: e0123369, 2015. doi:10.1371/journal.pone.0123369. 25961819 PMC4427300

[14] Tomizawa Y, Sekido Y, Kondo M, Gao B, Yokota J, Roche J, Drabkin H, Lerman MI, Gazdar AF, Minna JD. Inhibition of lung cancer cell growth and induction of apoptosis after reexpression of 3p21.3 candidate tumor suppressor gene SEMA3B. Proc Natl Acad Sci USA 98: 13954–13959, 2001. doi:10.1073/pnas.231490898. 11717452 PMC61148

[15] Shahi P, Wang CY, Chou J, Hagerling C, Gonzalez Velozo H, Ruderisch A, Yu Y, Lai MD, Werb Z. GATA3 targets semaphorin 3B in mammary epithelial cells to suppress breast cancer progression and metastasis. Oncogene 36: 5567–5575, 2017. doi:10.1038/onc.2017.165. 28581515 PMC5629104

[16] Cao Y, Szabolcs A, Dutta SK, Yaqoob U, Jagavelu K, Wang L, Leof EB, Urrutia RA, Shah VH, Mukhopadhyay D. Neuropilin-1 mediates divergent R-Smad signaling and the myofibroblast phenotype. J Biol Chem 285: 31840–31848, 2010. doi:10.1074/jbc.M110.151696. 20675371 PMC2951255

[17] Glinka Y, Prud’homme GJ. Neuropilin-1 is a receptor for transforming growth factor beta-1, activates its latent form, and promotes regulatory T cell activity. J Leukoc Biol 84: 302–310, 2008. doi:10.1189/jlb.0208090. 18436584 PMC2504713

[18] Ma H, Liu S, Li S, Xia Y. Targeting growth factor and cytokine pathways to treat idiopathic pulmonary fibrosis. Front Pharmacol 13: 918771, 2022. doi:10.3389/fphar.2022.918771. 35721111 PMC9204157

[19] West DC, Rees CG, Duchesne L, Patey SJ, Terry CJ, Turnbull JE, Delehedde M, Heegaard CW, Allain F, Vanpouille C, Ron D, Fernig DG. Interactions of multiple heparin binding growth factors with neuropilin-1 and potentiation of the activity of fibroblast growth factor-2. J Biol Chem 280: 13457–13464, 2005. doi:10.1074/jbc.M410924200. 15695515

[20] Schramek H, Sarkozi R, Lauterberg C, Kronbichler A, Pirklbauer M, Albrecht R, Noppert SJ, Perco P, Rudnicki M, Strutz FM, Mayer G. Neuropilin-1 and neuropilin-2 are differentially expressed in human proteinuric nephropathies and cytokine-stimulated proximal tubular cells. Lab Invest 89: 1304–1316, 2009. doi:10.1038/labinvest.2009.96. 19736548

[21] Meng Z, Li FL, Fang C, Yeoman B, Qiu Y, Wang Y, Cai X, Lin KC, Yang D, Luo M, Fu V, Ma X, Diao Y, Giancotti FG, Ren B, Engler AJ, Guan KL. The Hippo pathway mediates Semaphorin signaling. Sci Adv 8: eabl9806, 2022. doi:10.1126/sciadv.abl9806. 35613278 PMC9132450

[22] Lai YJ, Tsai FC, Chang GJ, Chang SH, Huang CC, Chen WJ, Yeh YH. miR-181b targets semaphorin 3A to mediate TGF-beta-induced endothelial-mesenchymal transition related to atrial fibrillation. J Clin Invest 132, 2022. doi:10.1172/JCI142548. PMC9246393 35775491

[23] Yombo DJK, Odayar V, Gupta N, Jegga AG, Madala SK. The protective effects of IL-31RA deficiency during bleomycin-induced pulmonary fibrosis. Front Immunol 12: 645717, 2021. doi:10.3389/fimmu.2021.645717. 33815402 PMC8017338

[24] Singh B, Kasam RK, Sontake V, Wynn TA, Madala SK. Repetitive intradermal bleomycin injections evoke T-helper cell 2 cytokine-driven pulmonary fibrosis. Am J Physiol Lung Cell Mol Physiol 313: L796–L806, 2017. doi:10.1152/ajplung.00184.2017. 28775096 PMC5792179

[25] Hardie WD, Korfhagen TR, Sartor MA, Prestridge A, Medvedovic M, Le Cras TD, Ikegami M, Wesselkamper SC, Davidson C, Dietsch M, Nichols W, Whitsett JA, Leikauf GD. Genomic profile of matrix and vasculature remodeling in TGF-alpha induced pulmonary fibrosis. Am J Respir Cell Mol Biol 37: 309–321, 2007. doi:10.1165/rcmb.2006-0455OC. 17496152 PMC1994231

[26] Hardie WD, Le Cras TD, Jiang K, Tichelaar JW, Azhar M, Korfhagen TR. Conditional expression of transforming growth factor-alpha in adult mouse lung causes pulmonary fibrosis. Am J Physiol Lung Cell Mol Physiol 286: L741–749, 2004. doi:10.1152/ajplung.00208.2003. 14660483

[27] Wang Y, Yella J, Chen J, McCormack FX, Madala SK, Jegga AG. Unsupervised gene expression analyses identify IPF-severity correlated signatures, associated genes and biomarkers. BMC Pulm Med 17: 133, 2017. doi:10.1186/s12890-017-0472-9. 29058630 PMC5649521

[28] Ghandikota S, Sharma M, Ediga HH, Madala SK, Jegga AG. Consensus gene co-expression network analysis identifies novel genes associated with severity of fibrotic lung disease. Int J Mol Sci 23: 5447, 2022. doi:10.3390/ijms23105447. 35628257 PMC9141193

[29] Furusawa H, Cardwell JH, Okamoto T, Walts AD, Konigsberg IR, Kurche JS, Bang TJ, Schwarz MI, Brown KK, Kropski JA, Rojas M, Cool CD, Lee JS, Wolters PJ, Yang IV, Schwartz DA. Chronic hypersensitivity pneumonitis, an interstitial lung disease with distinct molecular signatures. Am J Respir Crit Care Med 202: 1430–1444, 2020. doi:10.1164/rccm.202001-0134OC. 32602730 PMC7667907

[30] Adams TS, Schupp JC, Poli S, Ayaub EA, Neumark N, Ahangari F, Chu SG, Raby BA, DeIuliis G, Januszyk M, Duan Q, Arnett HA, Siddiqui A, Washko GR, Homer R, Yan X, Rosas IO, Kaminski N. Single-cell RNA-seq reveals ectopic and aberrant lung-resident cell populations in idiopathic pulmonary fibrosis. Sci Adv 6: eaba1983, 2020. doi:10.1126/sciadv.aba1983. 32832599 PMC7439502

[31] Dziobek K, Opławski M, Grabarek B, Zmarzły N, Januszyk P, Adwent I, Dąbruś D, Leśniak E, Kiełbasiński R, Kieszkowski P, Boroń D. Expression of semaphorin 3B (SEMA3B) in various grades of endometrial cancer. Med Sci Monit 25: 4569–4574, 2019. doi:10.12659/MSM.916762. 31217417 PMC6598462

[32] Sontake V, Shanmukhappa SK, DiPasquale BA, Reddy GB, Medvedovic M, Hardie WD, White ES, Madala SK. Fibrocytes regulate Wilms tumor 1-positive cell accumulation in severe fibrotic lung disease. J Immunol 195: 3978–3991, 2015. doi:10.4049/jimmunol.1500963. 26371248 PMC4592828

[33] Kasam RK, Reddy GB, Jegga AG, Madala SK. Dysregulation of mesenchymal cell survival pathways in severe fibrotic lung disease: the effect of nintedanib therapy. Front Pharmacol 10: 532, 2019. doi:10.3389/fphar.2019.00532. 31156440 PMC6533541

[34] Kasam RK, Gajjala PR, Jegga AG, Courtney JA, Randell SH, Kramer EL, Clancy JP, Madala SK. Fibrocyte accumulation in the lungs of cystic fibrosis patients. J Cyst Fibros 19: 815–822, 2020. doi:10.1016/j.jcf.2020.06.011. 32593509 PMC7492481

[35] Yombo DJK, Mentink-Kane MM, Wilson MS, Wynn TA, Madala SK. Heat shock protein 70 is a positive regulator of airway inflammation and goblet cell hyperplasia in a mouse model of allergic airway inflammation. J Biol Chem 294: 15082–15094, 2019. doi:10.1074/jbc.RA119.009145. 31431507 PMC6791332

[36] Christie SM, Hao J, Tracy E, Buck M, Yu JS, Smith AW. Interactions between semaphorins and plexin-neuropilin receptor complexes in the membranes of live cells. J Biol Chem 297: 100965, 2021. doi:10.1016/j.jbc.2021.100965. 34270956 PMC8350011

[37] Wynn TA. Cellular and molecular mechanisms of fibrosis. J Pathol 214: 199–210, 2008. doi:10.1002/path.2277. 18161745 PMC2693329

[38] Wynn TA, Ramalingam TR. Mechanisms of fibrosis: therapeutic translation for fibrotic disease. Nat Med 18: 1028–1040, 2012. doi:10.1038/nm.2807. 22772564 PMC3405917

[39] Moss BJ, Ryter SW, Rosas IO. Pathogenic mechanisms underlying idiopathic pulmonary fibrosis. Annu Rev Pathol 17: 515–546, 2022. doi:10.1146/annurev-pathol-042320-030240. 34813355

[40] Ma Y, Zhang Z, Chen R, Shi R, Zeng P, Chen R, Leng Y, Chen AF. NRP1 regulates HMGB1 in vascular endothelial cells under high homocysteine condition. Am J Physiol Heart Circ Physiol 316: H1039–H1046, 2019. doi:10.1152/ajpheart.00746.2018. 30767669

[41] Madala SK, Korfhagen TR, Schmidt S, Davidson C, Edukulla R, Ikegami M, Violette SM, Weinreb PH, Sheppard D, Hardie WD. Inhibition of the alphavbeta6 integrin leads to limited alteration of TGF-α-induced pulmonary fibrosis. Am J Physiol Lung Cell Mol Physiol 306: L726–735, 2014. doi:10.1152/ajplung.00357.2013. 24508732 PMC3989722

[42] Madala SK, Thomas G, Edukulla R, Davidson C, Schmidt S, Schehr A, Hardie WD. p70 ribosomal S6 kinase regulates subpleural fibrosis following transforming growth factor-α expression in the lung. Am J Physiol Lung Cell Mol Physiol 310: L175–L186, 2016. doi:10.1152/ajplung.00063.2015. 26566903 PMC4719051

[43] Madala SK, Edukulla R, Phatak M, Schmidt S, Davidson C, Acciani TH, Korfhagen TR, Medvedovic M, Lecras TD, Wagner K, Hardie WD. Dual targeting of MEK and PI3K pathways attenuates established and progressive pulmonary fibrosis. PloS one 9: e86536, 2014. doi:10.1371/journal.pone.0086536. 24475138 PMC3903543

[44] Li GZ, Shen D, Li GH, Wei M, Zheng LJ, Liu ZL, Sun RQ, Zhou SJ, Zhang ZL, Gao YC. Decreased expression of serum semaphorin 3B is associated with poor prognosis of patients with hepatocellular carcinoma. Exp Ther Med 21: 236, 2021. doi:10.3892/etm.2021.9667. 33603844 PMC7851624

[45] Pinezich MR, Tamargo MA, Fleischer S, Reimer JA, Hudock MR, Hozain AE, Kaslow SR, Tipograf Y, Soni RK, Gavaudan OP, Guenthart BA, Marboe CC, Bacchetta M, O’Neill JD, Dorrello NV, Vunjak-Novakovic G. Pathological remodeling of distal lung matrix in end-stage cystic fibrosis patients. J Cyst Fibros 21: 1027–1035, 2022. doi:10.1016/j.jcf.2022.04.016. 35525782 PMC10050894

[46] Luo H, Yan J, Zhou X. Constructing an extracellular matrix-related prognostic model for idiopathic pulmonary fibrosis based on machine learning. BMC Pulm Med 23: 397, 2023. doi:10.1186/s12890-023-02699-8. 37858084 PMC10585847

[47] Dong Z, Liang X, Wu X, Kang X, Guo Y, Shen S, Liang J, Guo W. Promoter hypermethylation-mediated downregulation of tumor suppressor gene SEMA3B and lncRNA SEMA3B-AS1 correlates with progression and prognosis of esophageal squamous cell carcinoma. Clin Exp Metastasis 36: 225–241, 2019. doi:10.1007/s10585-019-09964-3. 30915595

[48] Castro-Rivera E, Ran S, Thorpe P, Minna JD. Semaphorin 3B (SEMA3B) induces apoptosis in lung and breast cancer, whereas VEGF165 antagonizes this effect. Proc Natl Acad Sci USA 101: 11432–11437, 2004. doi:10.1073/pnas.0403969101. 15273288 PMC509218

[49] Zhao J, Bai J, Peng F, Qiu C, Li Y, Zhong L. USP9X-mediated NRP1 deubiquitination promotes liver fibrosis by activating hepatic stellate cells. Cell Death Dis 14: 40, 2023. doi:10.1038/s41419-022-05527-9. 36653359 PMC9849111

[50] Cao S, Yaqoob U, Das A, Shergill U, Jagavelu K, Huebert RC, Routray C, Abdelmoneim S, Vasdev M, Leof E, Charlton M, Watts RJ, Mukhopadhyay D, Shah VH. Neuropilin-1 promotes cirrhosis of the rodent and human liver by enhancing PDGF/TGF-beta signaling in hepatic stellate cells. J Clin Invest 120: 2379–2394, 2010. doi:10.1172/JCI41203. 20577048 PMC2898590

[51] Kang HR, Lee CG, Homer RJ, Elias JA. Semaphorin 7A plays a critical role in TGF-beta1-induced pulmonary fibrosis. J Exp Med 204: 1083–1093, 2007. doi:10.1084/jem.20061273. 17485510 PMC2118575

[52] Gan Y, Reilkoff R, Peng X, Russell T, Chen Q, Mathai SK, Homer R, Gulati M, Siner J, Elias J, Bucala R, Herzog E. Role of semaphorin 7a signaling in transforming growth factor beta1-induced lung fibrosis and scleroderma-related interstitial lung disease. Arthritis Rheum 63: 2484–2494, 2011. doi:10.1002/art.30386. 21484765 PMC3651701

[53] Peng HY, Gao W, Chong FR, Liu HY, Zhang JI. Semaphorin 4A enhances lung fibrosis through activation of Akt via PlexinD1 receptor. J Biosci 40: 855–862, 2015. doi:10.1007/s12038-015-9566-9. 26648031

